# From field to pond and beyond: bidirectional transport of pesticides and their transformation products in lentic small water bodies in Northern Germany

**DOI:** 10.1007/s11356-025-37317-z

**Published:** 2026-01-07

**Authors:** Lukas Paul Loose, Nicola Fohrer, Uta Ulrich

**Affiliations:** Department of Hydrology and Water Resources Management, Kiel University, Olshausenstraße 75, 24118 Kiel, Germany

**Keywords:** Ponds, Kettle holes, Groundwater, Drainage, Contamination pathways, Hydrological connectivity, Water quality

## Abstract

Lentic small water bodies (LSWBs) in agricultural landscapes are increasingly recognized for their ecological importance, but they remain vulnerable to agrochemicals, with pesticide contamination being of particular concern. This study investigated the spatio-temporal dynamics of 25 pesticides and their transformation products (TPs) in two LSWBs and their surrounding shallow groundwater in northern Germany. The aim was to identify dominant contamination pathways in LSWBs. Over two years, a high-resolution monitoring campaign collected pesticide concentration data on a daily to biweekly basis from surface water, shallow groundwater, and drainage inflows. Results revealed strong seasonal variations, with concentration peaks following pesticide applications and rainfall events. Metazachlor and flufenacet TPs dominated the compound spectrum due to their high mobility and persistence. Artificial drainage facilitated rapid pesticide transport and export, while shallow groundwater contributed to gradual, long-term inflows. Contamination patterns were shaped by the presence or absence of artificial drainage, groundwater inflow zones, and compound-specific behavior such as leaching potential and persistence. These findings emphasize the dual role of LSWBs as both sinks and sources of pesticide contamination and highlight the need to integrate subsurface and drainage pathways into monitoring and risk assessment frameworks of LSWBs.

**Supplementary Information:**

The online version contains supplementary material available at 10.1007/s11356-025-37317-z.

## Introduction

Lentic small water bodies (LSWBs), such as ponds and kettle holes, are permanent or seasonal, shallow pond ecosystems that typically range from 1 m^2^ to 5 ha in area (Biggs et al. 2017; Bolpagni et al. 2019; Oertli et al. 2005). These features are globally abundant, estimated to comprise about 8.6% of the world's total lake and pond surface area while only accounting for LSWB with a size of < 0.1 ha (Holgerson & Raymond 2016). LSWBs are a common landscape element worldwide. Particularly in Europe and North America, LSWBs are especially numerous and widespread across landscapes (Biggs et al. 2005; Kristensen & Globevnik 2014; Lorenz et al. 2017).

Despite their small size, they support rich and diverse communities of benthic invertebrates, aquatic plants, phytoplankton, and birds (Darwall et al. 2018; Dudzińska et al. 2020; Trau et al. 2023). These water bodies contribute to landscape-level biodiversity, ecological resilience, and overall watershed functionality (Bolpagni et al. 2019; Terasmaa et al. 2019). LSWBs provide a broad range of ecosystem services, including water retention, flood regulation, habitat provision, and biotic remediation of pollutants (Sender & Kułak 2014; Vasić et al. 2020). In agroecosystems, they offer key ecological functions for both aquatic and terrestrial species, serving as crucial refugia and stepping stones for biodiversity (Zamora-Marín et al. 2024).

Though they are a crucial landscape element, they often receive limited attention in global water body inventories (Bolpagni et al. 2019; Terasmaa et al. 2019) and are overlooked in environmental monitoring and regulatory frameworks, like the EU Water Framework Directive (European Commission 2000; Liess et al. 2023; Stanković et al. 2023). In addition, these systems are highly sensitive to anthropogenic pressures. Land use in the surrounding catchment, particularly the extent of arable land, has a strong influence on the integrity of LSWBs (Dudzińska et al. 2020; Joniak et al. 2016). Agricultural practices, including pesticide and fertilizer application, artificial drainage, and soil erosion, have been shown to degrade water quality and disrupt ecological balance (Kristensen & Globevnik 2014; Vasić et al. 2020; Willkommen et al. 2022). This persistent neglect, combined with multiple stressors, leads to severe contamination and poses direct and indirect threats to aquatic life and the overall ecosystem integrity within these vital habitats (Liess et al. 2023; Lorenz et al. 2025; Ulrich et al. 2022).

Numerous studies have detected a wide range of pesticides and their transformation products (TPs) in LSWBs and adjacent streams, with concentrations ranging from low ng L^−1^ to µg L^−1^ levels, occasionally exceeding regulatory thresholds (Betz-Koch et al. 2023; Navarro et al. 2024; Slaby et al. 2022). Concentrations often peak following applications and precipitation, indicating strong links between application timing, hydrological events, and pesticide mobility (Betz-Koch et al. 2023; Ulrich et al. 2018). While some systems show low contamination, others reveal ecotoxicologically relevant concentrations and mixture effects, particularly for aquatic organisms (Lorenz et al. 2025; Navarro et al. 2024). Importantly, detected residues may not stem solely from recent applications, but also from legacy inputs or long-range transport, highlighting the spatial and temporal complexity of pesticide fate in these vulnerable aquatic systems (Slaby et al. 2022; Ulrich et al. 2022).

Despite increasing recognition of their ecological value, LSWBs remain largely neglected in monitoring frameworks, resulting in a fragmented and unrepresentative data basis (Liess et al. 2023; Lorenz et al. 2017, 2025). Conventional sampling designs, based on weekly or monthly grab samples, often fail to capture short-term peak concentrations following pesticide application or rainfall events (Betz-Koch et al. 2023; Olsson et al. 2013; Szöcs et al. 2017). In particular, intensive agriculture introduces a major stressor, with pesticides entering LSWBs through various pathways such as drift, runoff, erosion, shallow groundwater (GW) exchange, and drainage inflow (Reichenberger et al. 2007). Yet, the relative contributions of these pathways remain poorly understood (Lischeid & Kalettka 2012; Ulrich et al. 2022). This uncertainty is further compounded by the limited inclusion of TPs in monitoring and regulatory schemes, despite their frequent detection, higher mobility, and often greater persistence compared to parent compounds (Gassmann et al. 2015; Le Cor et al. 2021; Pasquini et al. 2025). In addition, few studies track the full seasonal or interannual dynamics of pesticide contamination in LSWBs (Ulrich et al. 2018), and high-resolution, long-term monitoring efforts are still rare (Betz-Koch et al. 2023; Ulrich et al. 2018; Willkommen et al. 2022). This stands in contrast to the objectives of the European Union’s National Action Plans on the Sustainable Use of Pesticides (NAP), which explicitly call for the protection of small water bodies in agricultural landscapes (BMEL 2018; European Commission 2009). To close these gaps and provide better protection for small water bodies, high-resolution, long-term monitoring approaches that align with hydrological variability and pesticide application cycles are needed.

This study investigated the environmental fate of pesticides and their TPs in two LSWBs situated within an intensively cultivated lowland region in Northern Germany. A high-resolution monitoring campaign was conducted over multiple seasons, including daily to bi-weekly measurements of pesticide concentrations in surface water, shallow GW surrounding the ponds, and a tile drainage inflow connected to one of the sites. The study focuses on characterizing the concentrations and occurrence patterns of a broad set of pesticide compounds and their TPs. By integrating spatial and temporal data across different sampling locations and hydrological phases, the study seeks to identify dominant contamination pathways and understand how compound-specific properties and local hydrology shape pesticide transport and retention. The study thus provides a detailed case-based assessment of pesticide fate in LSWBs under conventional agricultural practice. The following research questions (RQ) were addressed:

- How do concentrations and detection amounts of pesticides and their transformation products vary spatially in LSWBs, shallow groundwater, and drainage? (RQ1)
- How do pesticide and TP concentrations vary temporally in the LSWBs and their adjacent environmental compartments? (RQ2)
- Which contamination pathways contribute most to pesticide and TP inputs and outputs in LSWBs, and which compounds dominate each of these pathways? (RQ3)

## Materials and methods

### Study area

**Fig. 1:**
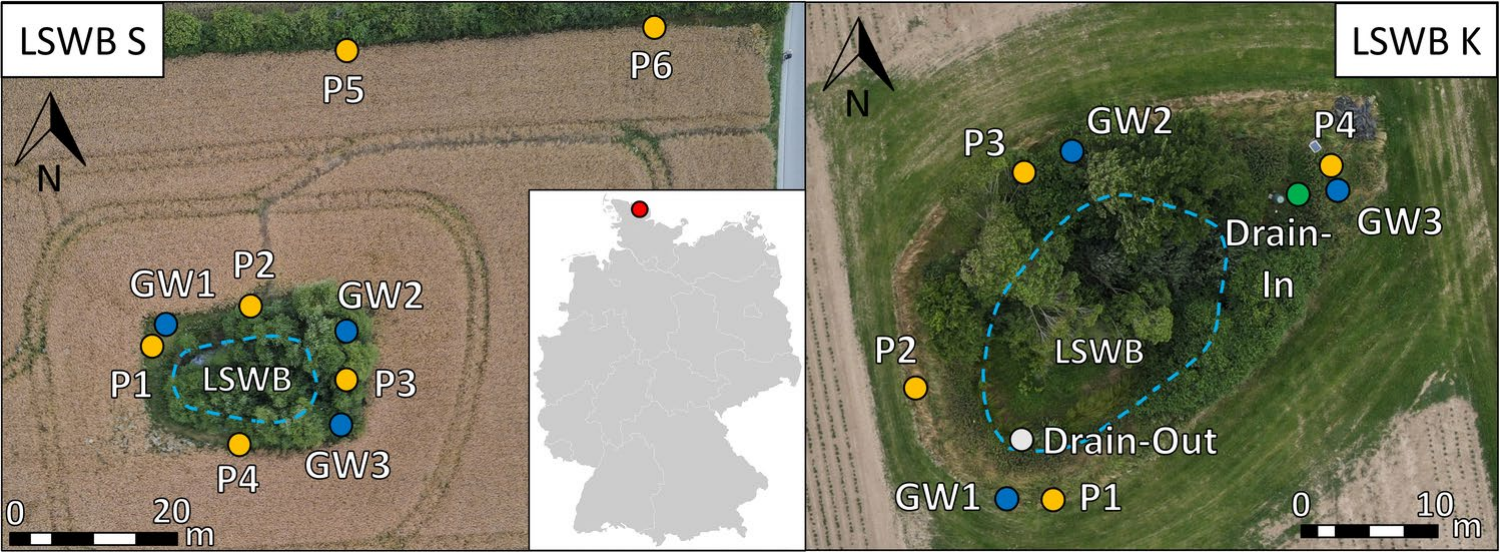
Map of Germany showing the sampling location within the Kielstau catchment in Schleswig-Holstein. Aerial images of the two lentic small water bodies (LSWB S and LSWB K) highlight the water areas as well as the piezometers (P), groundwater pipes (GW), and drainage inlet and outlet at LSWB K (Drain-In, Drain-Out) (adapted from Loose et al. 2024)

**Fig. 2:**
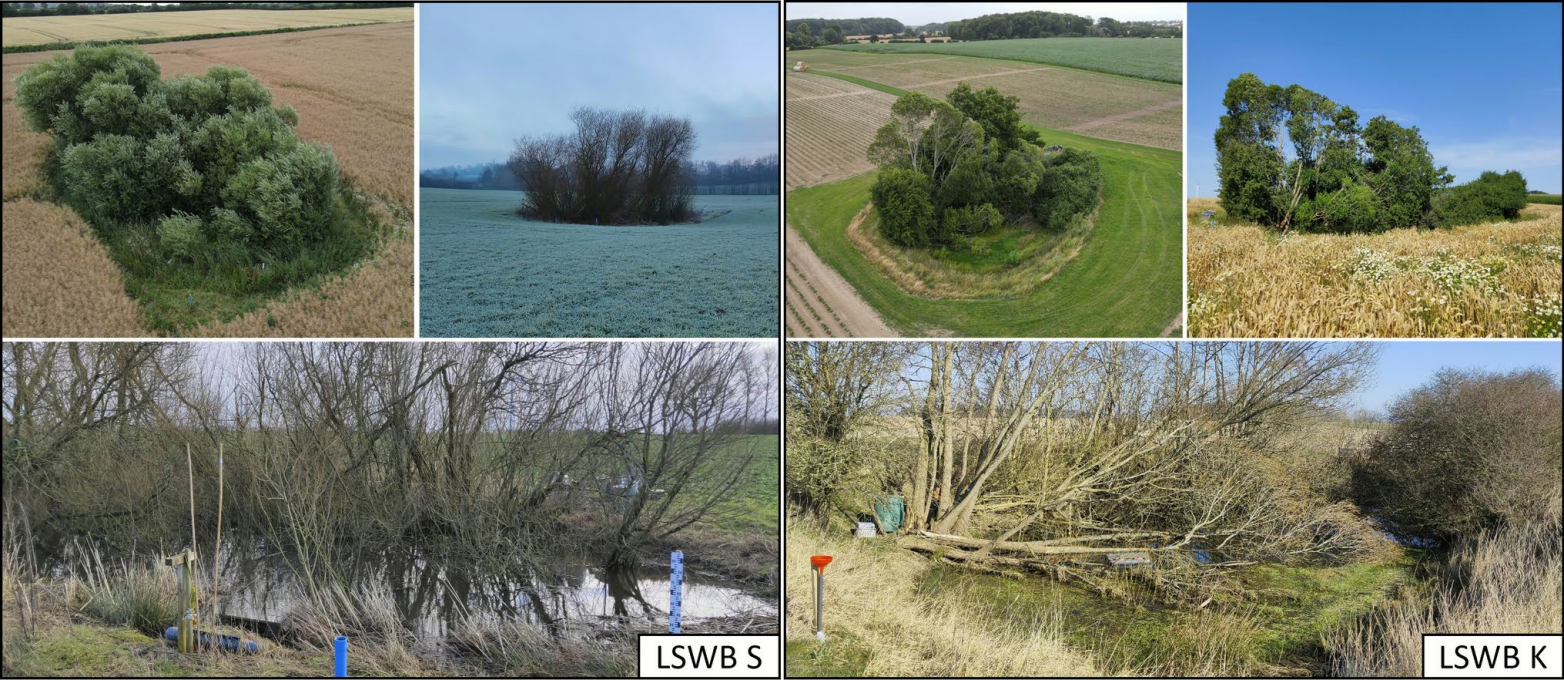
Field photographs of the two lentic small water bodies (LSWB S and LSWB K) in their agricultural setting. The photographs show the LSWBs in different seasons throughout the monitoring period. Labels indicate LSWB S and LSWB K

The study was conducted in the Kielstau catchment, a young moraine region in the lowlands of Northern Germany (Fig. 1). This landscape is characterized by intensive conventional agriculture, a low hydraulic gradient, and shallow GW conditions (Wagner et al. 2018). The average annual air temperature is 9.0 °C, and mean annual precipitation amounts to 890 mm, based on data from 1991 to 2022 (DWD 2024). The predominant soils in the study area are Planosols and Luvisols in the higher lying areas, while Gleysols and Histosols are typical for lower bottomlands (Wagner et al. 2018). The two LSWBs, referred to as LSWB K and LSWB S (Fig. 2, Table 1), are each surrounded by arable fields under conventional management and are bordered by strips of grassland, bushes, and trees. During the 2020–2021 growing season, winter barley (WB) was cultivated around both LSWBs. After harvest, the field around LSWB K was leased and only catch crops were grown without further pesticide application. In contrast, the area around LSWB S was planted with winter oilseed rape (WOR), which was cultivated until 2022. Around both ponds, agricultural tillage created ploughed furrows along the field–pond boundary that prevented direct surface runoff from the surrounding fields.

**Table 1:** Characteristics of LSWB S and LSWB K. *LSWB elevation at 0 cm water level, **at 1 m water level, ***depth of water body and organic mud layer until the beginning of dense clay mud at 1 m water level, ****depth of the water body above the mud layer (adapted from Loose et al. 2024)

Characteristics	LSWB S	LSWB K
Coordinates	54°44.125680′N, 9°39.917340′O	54°45.035880′N, 9°41.409180′O
Altitude (m a.s.l.)	48.45*	45.76*
Water surface area (m^2^)	264.13**	271.18**
Circumference (m)	57.61**	58.38**
Total depth (m)	4.85***	5.24***
Water level average (m)	0.59	0.82
Water level max. (m)	1.09****	1.20****
Water level amplitude (m)	1.09	0.83
Length to wide ratio	1.74:1	1.94:1
Surrounding soil types	Stagnisols, Planosols, Gleysols	Stagnisols, Planosols, Gleysols
Soil texture	loamy/silty sand, sandy silt	loamy/silty sand

The hydrological conditions of the two LSWBs differ. LSWB K is both fed and drained via subsurface drainage pipes, while LSWB S lacks artificial inflow or outflow and regularly dries out during summer. Yet both LSWBs are highly influenced by the shallow GW. LSWB K exhibited a higher density of aquatic vegetation, including algae and macrophytes, compared to LSWB S. To investigate shallow GW at different depths, three groundwater pipes (GW1–GW3) and four shorter groundwater pipes, hereafter referred to as “piezometers” (P1–P4), were installed around the two LSWBs. Two additional piezometers (P5 and P6) were installed in the presumed inflow zone at LSWB S, located at a greater distance. Piezometer filters were at a depth of 1.5–2 m below surface, and filters of the GW pipes reached down to 3 m. All filters were surrounded by gravel, and the locations of all installations were recorded using a GPS device (Leica Geosystems GS09).

### Sampling campaign

The sampling campaign was conducted between the 1 st of September 2020 and 4^th^ of August 2022. Both LSWBs and the drainage inlet (Drain-In) at LSWB K were equipped with automatic water samplers (Teledyne ISCO 6700). Ten subsamples were collected per day at equal time intervals and combined into a single daily composite sample. Pond water samples were taken from the center of each water body, while drainage samples were collected directly from the pipe. Manual grab samples were collected weekly from the piezometers and biweekly from the GW pipes using a custom-built pump. Prior to sampling, standing water in the pipes was purged, and the freshly recharged water was used for analysis. In total, 941 samples were collected at LSWB S and 1485 samples at LSWB K. In-situ water quality parameters, including temperature, dissolved oxygen, pH, redox potential, and electrical conductivity, were automatically measured in both water bodies using UIT Sensodive Multi-Sensor Modules. The same parameters were recorded in freshly recharged water from piezometers and GW pipes using a WTW multimeter (3430). Precipitation data were obtained from the DWD weather station in Wagersrott at a 10 km distance (DWD 2024). Additionally, the hydrology of both LSWB systems was assessed. Further details of these investigations are provided in Loose et al. (2024). 

### Selected pesticides, transformation products, and analysis

#### Properties and application dates

A total of 25 compounds, including herbicides, fungicides, insecticides, and their TPs, were selected for analysis (Table 2). The selection was based on prior consultation with local farmers to include compounds planned for application during the study period. In addition, commonly used and frequently detected pesticides and TPs were incorporated to ensure a comprehensive assessment of potential contamination. The selected compounds reflect a wide range of properties. These include organic carbon-normalized Freundlich sorption coefficients (K_foc_), which describe non-linear sorption behavior; the Groundwater Ubiquity Score (GUS) as an indicator of the leaching potential, with GUS values < 1.8 indicating “unlikely to leach”, 1.8–2.8 “marginal leaching potential”, and > 2.8 “likely to leach” (Gustafson 1989); and dissipation half-lives under field conditions (DT_50_ field (Lewis et al. 2016)). Application dates and quantities were also provided by the farmers (Table 3).

**Table 2:** Selected pesticides with the soil adsorption coefficient (K_foc_ or Koc, mL g^−1^), field dissipation half-life (DT_50_ field, days), water dissipation half-file (DT_50_ water, days), and GUS leaching potential index based on Lewis et al. (2016). Lab-derived DT_50_ values are marked with (l), and values based on Koc rather than K_foc_ are marked with (k_oc_), if no field data or K_foc_ were available

Compounds	K_foc_ (mL g^−1^)	DT_50_ field (d)	DT_50_ water (d)	GUS leaching potential index
Bixafen	3869	254	26	Unlikely
Diflufenican	2215	94.5	-	Marginal
Dimethenamid	69	13	-	Marginal
Florasulam	20.37	8.5	54	Marginal
Flufenacet	221.25	35.7	29.35	Marginal
Flufenacet-ESA	12.5	302 (l)	-	Likely
Flufenacet-OA	10.6	5.83 (l)	-	Marginal
Fluroxypyr	68	3	10.5	Unlikely
Mefenpyr-diethyl	615	17.5 (l)	80	Unlikely
Mesosulfuron	68.3	39.7	-	Likely
Mesotrione	83.3	5	5.3	Unlikely
Metazachlor	79.6	6.8	216	Unlikely
Metazachlor-ESA	9 (k_oc_)	400	-	Likely
Metazachlor-OA	24.6	96.3	-	Likely
Metconazole	1116	134.7	8	Marginal
Metolachlor	163	21	88	Marginal
Metrafenone	3105	62	3.9	Unlikely
Norflurazon	295	225	-	Likely
Pendimethalin	13,792	100.6	4	Unlikely
Pirimicarb	166.8	9	22	Unlikely
Propoxycarbazone	32	5.5	50.7	Marginal
Prothioconazole	2556	0.77	0.37	Unlikely
Quinmerac	86	9.8	88.7	Marginal
Tebuconazole	769	47.1	42.6	Marginal
Terbuthylazine	231	21.8	-	Marginal
Terbuthylazine-desethyl	78	28.6	6	Likely
Triclopyr	57.95	30	24.8	Likely

**Table 3:** Application data at LSWB S and K. The crops were winter barley (WB) and winter oilseed rape (WOR). The columns ‘Application date(s)’ and ‘Application amount’ specify where and when each pesticide was applied, and at what rate

Compounds	LSWB S			LSWB K		
	Crop	Application date(s)	Application amount (g ha^−1^)	Crop	Application date(s)	Application amount (g ha^−1^)
**Bixafen**	WB	Jun 21	52	-	-	-
**Diflufenican**	WB	Sep 20	90–120	WB	Sep 20	120
**Florasulam**	WB, WOR*	Jun 20, Apr 21	1.9–5	-	-	-
**Flufenacet**	WB	Sep 20	120–160	WB	Sep 20	120
**Metazachlor**	WOR*	Aug 21	675	-	-	-
**Metconazole**	WOR*	Oct 21	12	-	-	-
**Pendimethalin**	WB, WOR*	Sep 20, Nov 21	600–1000	-	-	-
**Prothioconazole**	WB	Apr 21, May 21, Jun 21	72–120	WB	May 21, Jun 21	72–120
**Quinmerac**	WOR*	Aug 21	225	-	-	-
**Tebuconazole**	WB	Oct 20, Sep 21, Oct 21, Apr 22	62.5–150	WB	Oct 20	62.5

#### Solvents and chemicals

Methanol (MeOH, ≥ 99.9%, LC–MS grade, Sigma Aldrich), ultra-pure water (PURELAB flex, ELGA LabWater), calcium chloride (97%, Thermo Fisher Scientific), ammonium formate (> 99%, LC–MS grade, VWR), and formic acid (98–100%, LC–MS grade, Sigma Aldrich) were used as solvents and for standard preparation. Pesticide standards for spiking, internal standards (ISTD), and calibration solutions (100 µg/mL in acetonitrile) were obtained from HPC Standards GmbH and Neochema GmbH with a clarity of ≤ 100%.

#### Lab analysis

Water samples were analyzed by direct injection without prior filtration or extraction. After sampling, 1 mL aliquots of the unfiltered water samples were transferred into autosampler vials and stored cooled and in the dark until analysis. Multi-residue pesticide analysis was performed using an Agilent 6495B LC–MS system equipped with a reverse-phase C18 column (Phenomenex Synergi, 4 µm, Hydro-RP 80 Å, 150 × 3 mm). The mobile phase consisted of methanol and ultra-pure water that was modified with 0.005% formic acid and 2.5 mmol L^−1^ ammonium formate, applied via gradient elution. Limits of detection (LOD) and quantifications (LOQ) ranged between 0.0015–0.025 µg L⁻^1^ and 0.005–0.075 µg L⁻^1^ (see supplementary information for detailed values). Internal standards were added automatically into the samples during injection: D6-metazachlor-ESA at 1.5 µg L⁻^1^ and D5-terbuthylazine at 0.5 µg L⁻^1^. Calibration was carried out using an eight-point curve spanning concentrations from 0.005 to 1 µg L⁻^1^, freshly generated by the autosampler at the beginning of each sequence. All calibration curves achieved correlation coefficients above 0.99 (*R*^2^).

To verify accuracy and recovery, every fifth sample was spiked with 0.5 µg L⁻^1^ of the target analytes and analyzed together with the unspiked samples. Additionally, standard solutions at 0.25 µg L⁻^1^ were regularly measured to monitor instrument performance and reproducibility. Procedural blanks were included after each spike and standard to control for potential contamination and ensure analytical consistency. Compound identification was based on matching precursor–product ion transitions and retention times with those of analytical standards. Chromatograms were manually inspected to confirm signal identity and rule out matrix interferences. Quality control for all analyzed compounds, including reproducibility, recovery, and repeatability, is summarized in the supplementary information (Table 9). Detailed LC-MS parameters are also provided with the supplementary information.

#### Data analysis

Values below the limits of detection (LOD) and limits of quantification (LOQ) were excluded from further data analysis to prevent bias from non-detectable concentrations.

To enable realistic detection amounts, continuous concentration and mass flux calculations were performed despite discrete sampling, using linear interpolation for pesticide and TP concentrations under specific conditions. For GW, interpolation was conducted when two sampling points were no more than 15 days apart. For piezometers, interpolation was applied when the time between consecutive samples did not exceed 8 days. Interpolation was only performed when both sampling dates fell within periods when the GW or piezometer pipes were confirmed to contain water and when compounds were detected above the LOQ at both time points. This approach assumes that pesticide and TP concentrations were likely present between measurements, allowing for a more complete estimation of contaminant dynamics during unsampled periods.

The total daily mass of pesticides and TPs in each LSWB was estimated by multiplying the respective daily LSWB volume (m^3^) by the total concentrations of all compounds (µg L^−1^). Daily inflow loads were calculated by multiplying the inflow volume from either piezometers or GW into the LSWB by the corresponding pesticide and TP concentrations. Daily outflow loads were determined by using the outflow volume from the LSWB into the surrounding piezometers or GW pipes by multiplying the pesticide and TP concentrations in the LSWB. All hydrological calculations can be found in Loose et al. (2024). The hydrological phases of the LSWBs, specifically the filling periods, plateau phases with relatively stable water levels, and drying periods, were classified for the entire sampling period using the approach outlined in Loose et al. (2024).

Data processing and visualization were conducted in Python (v3.11.11), utilizing the Matplotlib and Seaborn libraries for plotting. Microsoft Excel was used alongside for data organization, manual inspection, and specific descriptive analyses.

## Results

### Compound specific contamination patterns across sampling locations

**Fig. 3:**
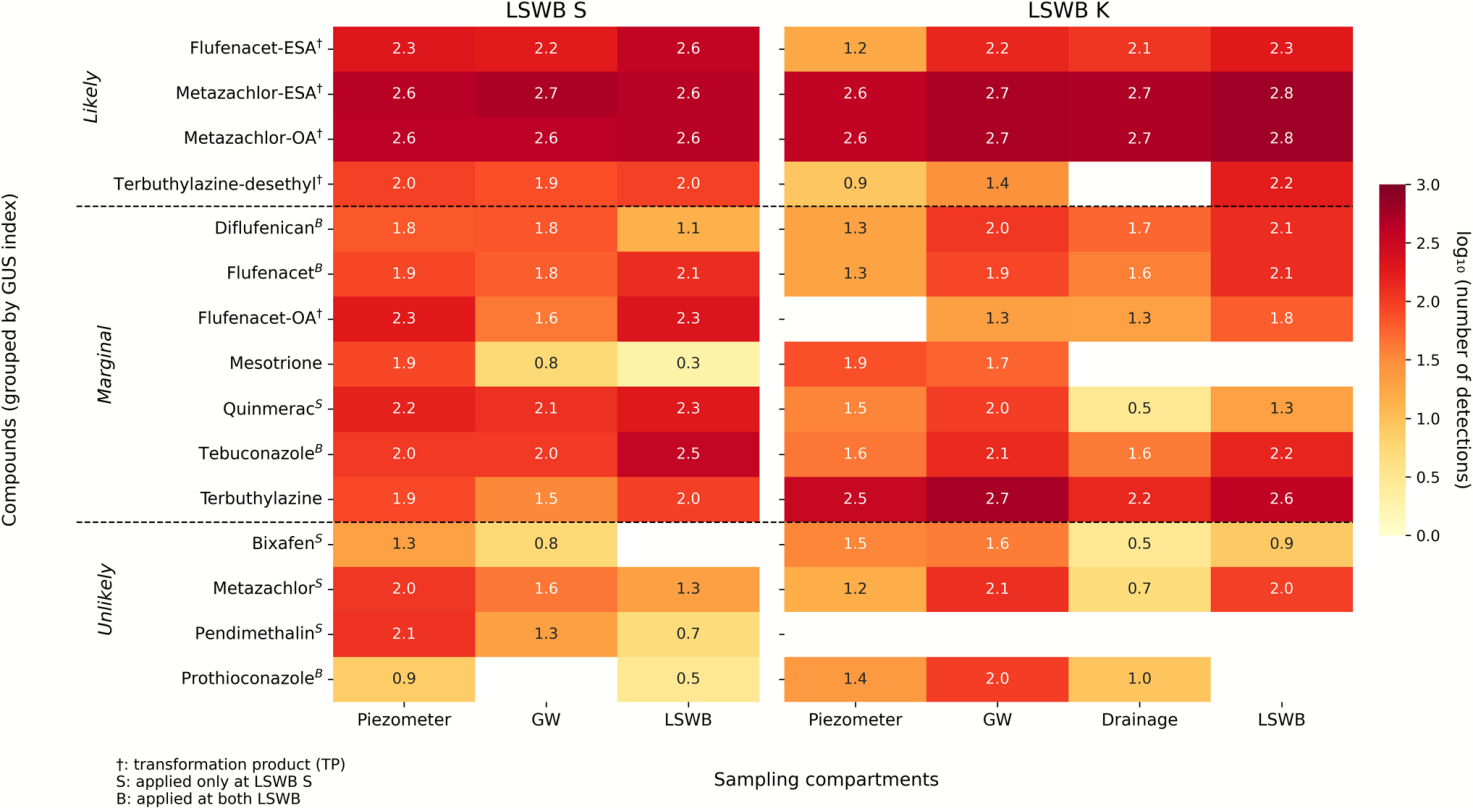
Detection amounts of detected compounds across all sampling locations for LSWB S and K and grouped according to their GUS index. The heatmap shows compound-wise detection amounts differentiated by location types: piezometers, groundwater (GW) pipes, drainage at LSWB K, and pond water (LSWB). Detection amounts for piezometers and groundwater pipes were calculated per single pipe by dividing total values by the number of respective installations (LSWB S: piezometer *n* = 2635, GW *n* = 1546, LSWB *n* = 490; LSWB K: piezometer* n* = 1159, GW *n* = 1586, drainage *n* = 618, LSWB = 708). Color intensity reflects detection amounts on a log-transformed scale, from low (light) to high (dark). Compounds marked with “†” indicate TPs, with “S” that were only applied at LSWB S, and with “B” that were applied at both LSWBs. Only compounds with detections > LOQ are displayed

To investigate how compound mobility influences environmental distribution, detection amounts (DAs) of selected pesticides and their TPs were analyzed across surface water, shallow GW pipes, piezometers, and artificial drainage at LSWB S and LSWB K (Fig. 3). Compounds were grouped by GUS leaching potential index to distinguish differences in transport behavior.

Compounds classified as "likely to leach" showed the highest DA across all sampling locations and dominated the overall detection frequencies throughout the sampling period. This class included the largest number of TPs. Notably, metazachlor-ESA and metazachlor-OA were detected in over 90% of all samples at both LSWB S and LSWB K, despite the parent compound only being applied at LSWB S during the study period. These TPs also reached the highest concentrations of the entire monitoring period, with maxima of 19.7 and 10.7 µg L⁻^1^, respectively (Table 4). Terbuthylazine-desethyl, a TP of terbuthylazine, was present system-wide despite no recorded application of its parent compound.

**Table 4:** Maximum (max.) and mean concentrations of findings > LOQ across all sampling locations at LSWB S and K for detected pesticides and TPs

GUS leaching potential	Compound	LSWB S		LSWB K	
		**Max. concentration (µg L**^**−1**^**)**	**Mean concentration (µg L**^**−1**^**)**	**Max. concentration (µg L**^**−1**^**)**	**Mean concentration (µg L**^**−1**^**)**
**Likely to leach**	Flufenacet-ESA	5.329	0.125	1.500	0.227
	Metazachlor-ESA	19.721	2.780	11.638	1.787
	Metazachlor-OA	10.774	0.917	9.679	0.621
	Terbuthylazine-desethyl	0.048	0.010	0.172	0.012
**Marginal leaching potential**	Diflufenican	5.404	0.121	5.539	0.110
	Flufenacet	0.355	0.012	0.194	0.027
	Flufenacet-OA	0.848	0.038	0.303	0.080
	Mesotrione	1.072	0.083	2.881	0.194
	Quinmerac	0.662	0.024	0.077	0.015
	Tebuconazole	1.089	0.016	0.486	0.025
	Terbuthylazine	0.017	0.010	0.045	0.014
**Unlikely to leach**	Bixafen	0.012	0.008	0.016	0.009
	Metazachlor	0.159	0.023	0.152	0.036
	Pendimethalin	0.244	0.022	0.019	0.01
	Prothioconazole	0.021	0.01	13.835	2.099

Compounds with marginal leaching potential showed lower DAs and concentrations overall (Table 4). This group mainly comprised applied compounds, except for mesotrione and terbuthylazine, and only one TP, flufenacet-OA. Flufenacet, applied at both LSWBs, showed moderate DA but lower concentrations than its TPs. Additionally, in this group a depth-related trend emerged, with higher DA observed in shallow piezometers and GW compared to the deeper pond, especially for diflufenican and mesotrione at LSWB S. At LSWB K, however, the highest DA occurred in GW, followed by pond water and piezometers, with only few DA in the drainage. Notably, as shown in Fig. 3, diflufenican was detected in drainage water at LSWB K, despite its relatively high K_foc_. Tebuconazole, applied multiple times, was detected in moderate amounts across all sampling points with peaking concentrations after the applications and overall low concentrations (Table 4).

Compounds considered unlikely to leach, characterized e.g. by high K_foc_ values, generally showed low DA. Their presence was mostly limited to shallow piezometers, GW and drainage at LSWB K, with minimal detection in surface water. Bixafen was detected across all compartments at LSWB K in low concentrations, despite no recorded application. Pendimethalin and prothioconazole appeared sporadically at LSWB S in low amounts following application with peaking concentrations, reflecting their strong sorption behavior and limited mobility.

At LSWB S, consistently high DA across all sampling locations reflects a more uniform and widespread contamination pattern throughout the system. In contrast, at LSWB K, elevated DA in GW and drainage water points to these sampling locations as key sources contributing to surface water contamination. The particularly high DA of “likely to leach” compounds in the drainage highlights the role of artificial drainage systems in facilitating pesticide transport.

### Seasonal trends and spatial gradients of pesticide and TP concentrations

**Fig. 4:**
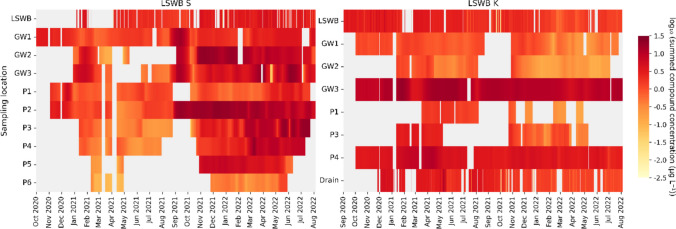
Temporal dynamics of total pesticide concentrations across all sampling locations at LSWB S and LSWB K. The heatmap shows the daily concentration of the sum of all analyzed pesticides and TPs > LOQ for each sampling location throughout the monitoring period. Color intensity reflects concentration on a log-transformed scale, with darker shades indicating higher total concentrations. Sampling locations include surface water (LSWB), piezometers (P), groundwater (GW) pipes, and drainage (Drain, LSWB K only). Grey areas indicate no data, either because of dry conditions, no measured concentration, or no samples taken

To assess spatial and temporal pesticide dynamics, daily total concentrations of all analyzed pesticides and TPs were visualized per sampling location throughout the monitoring period (Fig. 4). This representation highlights both seasonal concentration peaks and spatial differences across pond water, piezometers, GW pipes, and drainage.

At LSWB S, overall pesticide concentrations were higher than at LSWB K. During the first half of the monitoring period, low water levels limited data availability and concentrations remained low. In the second half, higher water levels in the pond and adjacent compartments coincided with increased concentrations, particularly in autumn and winter, aligning with the main pesticide application period. Spatially, contamination patterns varied across piezometers and GW pipes. In the outflow area, GW1 showed the lowest mean concentrations (2.66 µg L⁻^1^), likely due to dilution by pond water. In contrast, GW2 and GW3, located in the inflow zone, reached higher values, with GW2 peaking at 8.16 µg L⁻^1^. Among piezometers, P1 in the outflow area showed low values, while P2–P4 on the inflow side exhibited elevated concentrations, particularly in the second half of the sampling period. P5 showed increasing levels from November 2021 onward, while P6, at the field edge, consistently had the lowest concentrations.

At LSWB K, concentrations were lower and declined over time. During the first half of the monitoring period, when pesticides were used, peak concentrations were observed across compartments. Pond concentrations gradually declined with the cessation of pesticide applications. The drainage system followed a similar trend, reflecting reduced inputs from the uncultivated field. Mean summed concentrations in the pond and drainage were comparable at 2.25 and 2.32 µg L⁻^1^. GW pipe dynamics varied within LSWB K. GW1 and GW2 consistently showed low concentrations, while GW3 had the highest mean summed concentration at 10.81 µg L⁻^1^. Among piezometers, P1 and P3 contained water only sporadically and had low concentrations. P4 retained water longer and showed elevated values similar to GW3.

The piezometers and GW pipes at LSWB K displayed considerable variation in concentration dynamics, similar to those at LSWB S, underscoring the importance of shallow GW transport and drainage systems in shaping local pesticide contamination patterns. Temporal variability was particularly pronounced in pond water and shallow piezometers, which responded more rapidly to application events, while deeper GW showed more attenuated concentration changes over time. Finally, the spatial pattern of highest concentrations in inflow area sampling locations, followed by lower concentrations in the pond, and further decreasing levels at the outflow, suggests a retention effect within the LSWBs.

### Contaminant transport pathways into and out of LSWBs

To understand the dominant contamination pathways and the temporal dynamics of pesticide transport, a mass balance approach was applied for both LSWBs. This involved quantifying water fluxes and pesticide loads entering and leaving the pond, as well as evaluating in-pond concentrations and total mass over time. The analysis was structured around hydrological phases—filling, plateau, and drying—which reflect distinct water level dynamics and associated transport processes.

#### Pesticide loads and phase-specific transport dynamics in LSWB S

**Fig. 5:**
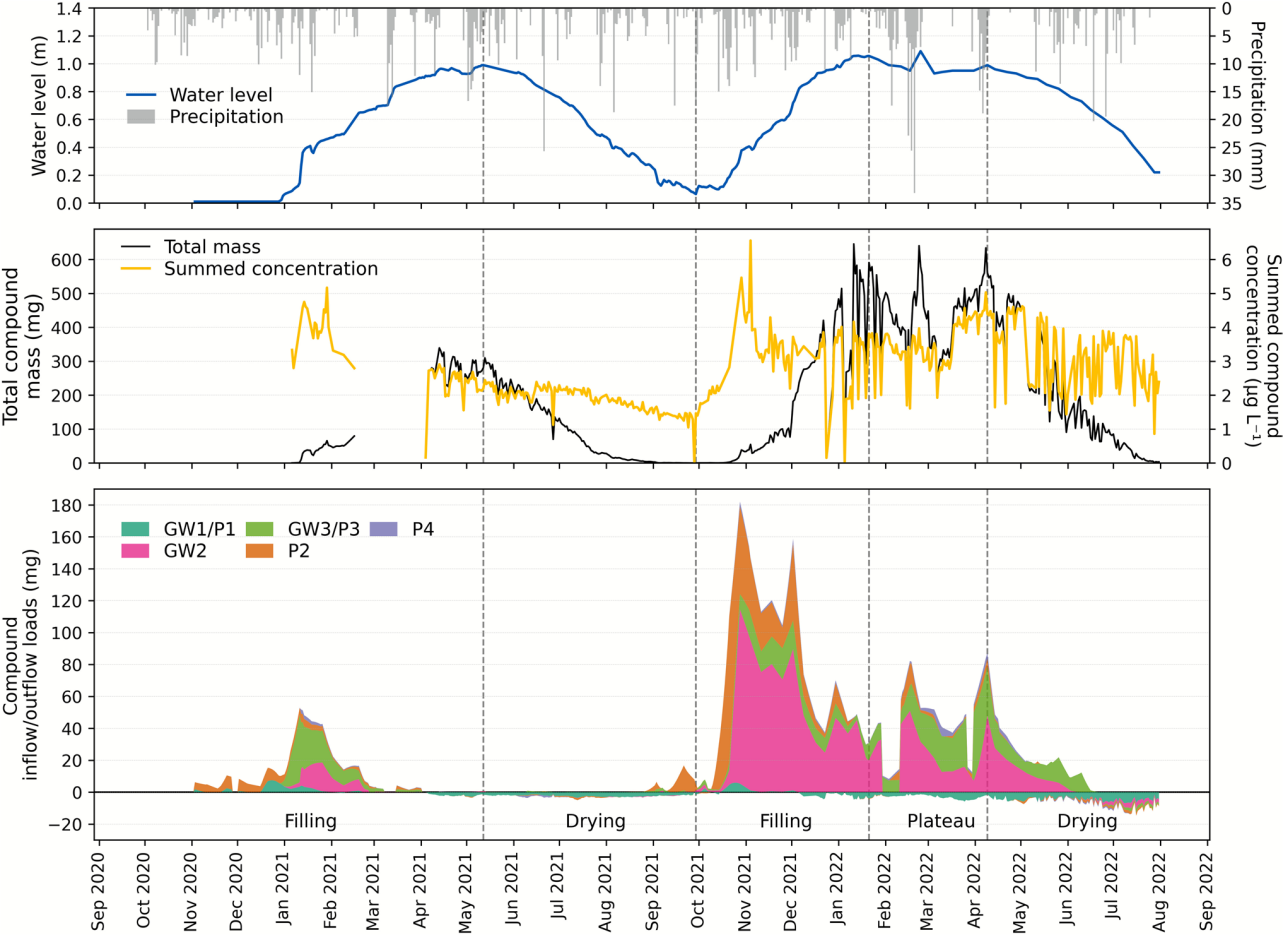
Hydrological and pesticide mass dynamics in LSWB S across the monitoring period. The upper part of the figure shows daily precipitation and pond water level. The middle section displays total pesticide concentrations in pond water alongside the calculated total pesticide mass in the pond. The lower section illustrates daily pesticide loads entering (positive) and leaving (negative) the pond via different pathways, including groundwater pipes (GW) and piezometers (P). Color scheme adapted to LSWB S for visual clarity

Figure 5 shows the temporal dynamics of hydrological conditions and pesticide contamination in LSWB S, including water level, precipitation, total pesticide concentration, total pesticide mass, and daily inflow and outflow loads. The pond exhibited two distinct filling phases, a drying phase, and a short plateau phase in 2022. This combined representation enables the identification of critical periods for input, retention, and loss of pesticides in relation to hydrological changes and pesticide application timing.

The first filling phase (2020/2021) was prolonged due to a dry summer and autumn in 2020, with the pond only reaching maximum water levels by mid-May 2021. Initial pesticide concentrations were already elevated at approximately 5 µg L⁻^1^ during the short sampling window from January to mid-February 2021 and increased in parallel with rising water levels. From April onwards, concentrations and mass remained stable. Inflow loads were recorded from all piezometers and GW pipes, with GW3/P3 contributing the most. As water levels continued to rise, outflow via GW1/P1 began in April, initiating the first export of water and solutes. The second filling phase (2021/2022) followed a wetter summer with elevated shallow GW levels. This led to a more dynamic inflow situation, dominated by inputs from GW2, followed by P2 and again GW3/P3. As a result, summed pesticide concentrations in the pond peaked at approximately 6.5 µg L^−1^. With the continued rise of pond water level, the hydraulic gradients gradually flattened, leading to a reduction in inflow loads over time.

The drying phases were characterized by a steady decline in water levels, accompanied by increasing outflow loads primarily via GW1/P1, but also from other piezometers and GW pipes to a lesser extent. As a consequence of water loss and pesticide export, the total pesticide mass and summed concentrations in the pond decreased during this period. A short plateau phase was observed in 2022, during which relatively stable water levels were maintained due to continued inflow from surrounding compartments. Despite the stable water level, summed pesticide concentrations in the pond continued to rise, while total pesticide mass fluctuated. Inflow loads remained substantial and variable until the end of the phase, indicating continued input from the surrounding shallow GW system.

These observations show that LSWB S is strongly influenced by GW interactions, particularly during filling and plateau phases. High concentrations and dynamic load patterns underline the role of shallow GW as a key contamination pathway into the pond, while the outflow-dominated drying phase facilitates contaminant export under decreasing water levels. Together, these observations reflect the strong interplay between hydrological conditions and pesticide mass balances. During filling and plateau phases, ponds may function as buffers and temporary sinks for pesticides, whereas during the drying phase, they can act as sources, as observed at LSWB S.

#### Pesticide loads and phase-specific transport dynamics in LSWB K

**Fig. 6:**
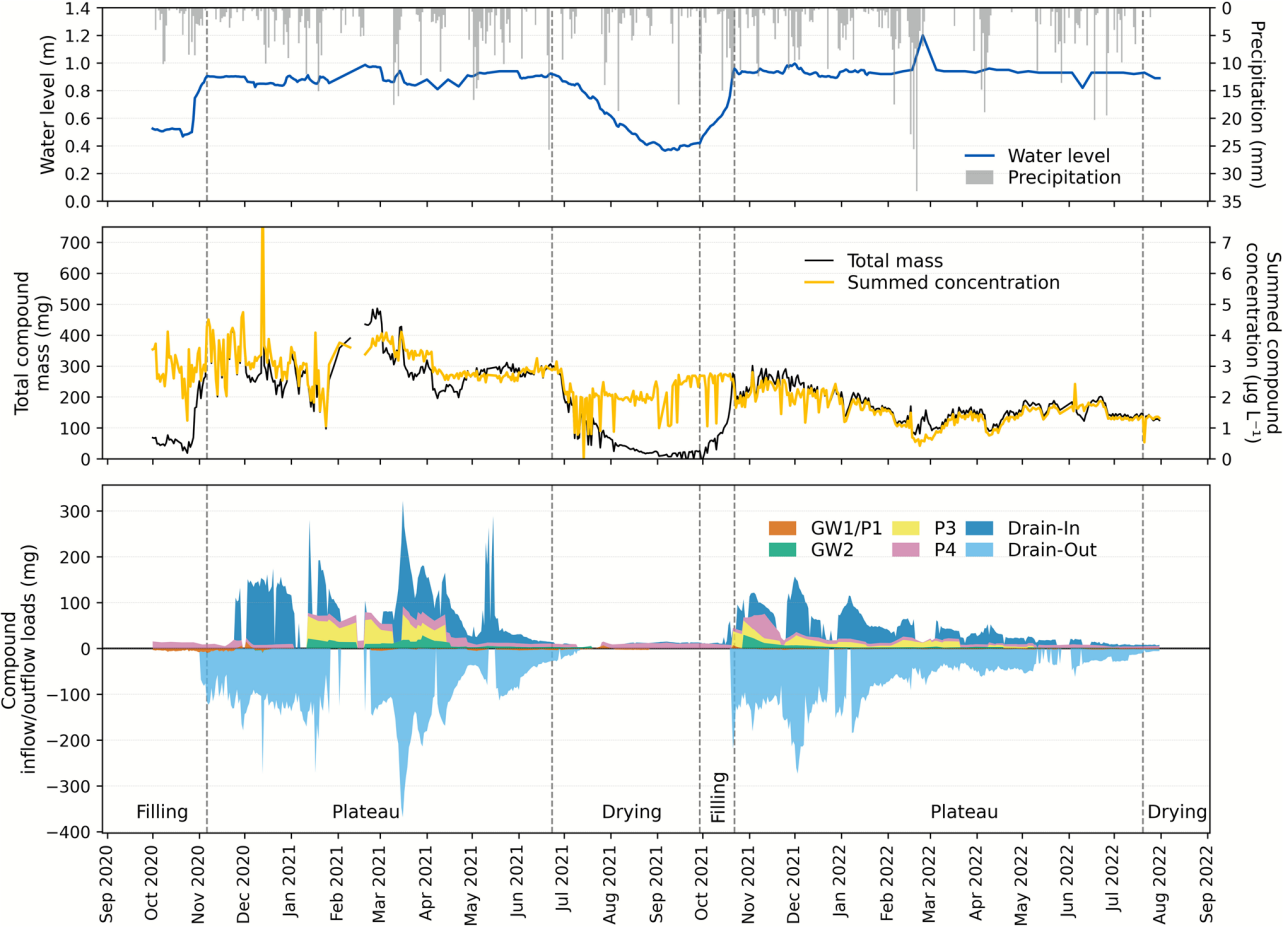
Hydrological and pesticide mass dynamics in LSWB K across the monitoring period. The upper part of the figure shows daily precipitation and pond water level. The middle section displays total pesticide concentrations in pond water alongside the calculated total pesticide mass in the pond. The lower section illustrates daily pesticide loads entering (positive) and leaving (negative) the pond via different pathways, including groundwater pipes (GW), piezometers (P), and the drainage inlet and outlet (Drain-In, Drain-Out). Color scheme adapted to LSWB K for visual clarity

Figure 6 displays hydrological and pesticide dynamics in LSWB K across two full annual cycles, each comprising a filling, plateau, and drying phase. It integrates precipitation and water level, total pesticide concentrations and in-pond mass, and inflow/outflow load fluxes.

Filling phases were short, marked by rapid increases in pond water level after dry periods. These phases coincided with key pesticide application events, but inflow loads remained low. Limited pesticide input during this time resulted from weak hydraulic gradients and restricted exchange with shallow GW in the observed 3 m depth, as water tables were still recovering. Although total pesticide concentrations in the pond remained low, the total pesticide mass increased in parallel with rising water volume. Exchange with surrounding compartments, especially piezometer P4 and GW pipe GW1/P1, was minimal and remained relatively stable across the phase.

Plateau phases were characterized by stable pond levels sustained by continuous drainage outflow once a threshold level was reached. In 2021, pesticide concentrations increased until mid-December due to application-related inputs and infiltration. Concentrations then declined until mid-January, before a temporary increase in early spring driven by shallow GW contributions. Total pesticide mass followed concentration and water level dynamics, peaking in March 2021. The drainage inlet dominated inflows during rainfall events, while GW contributions were most relevant between January and April. Outflows through the drainage outlet were steady and exported most of the pond’s pesticide load. In contrast, the 2022 plateau phase showed lower pesticide concentrations and mass overall. Inputs from shallow GW and the drainage inlet declined due to cessation of pesticide use and lower hydrological activity, consistent with the non-cultivation of the adjacent field. Outflow volumes were also reduced, leading to gradual contaminant attenuation within the pond.

Finally, the drying phases in both years were dominated by declining water levels. This was accompanied by a reduction in total pesticide mass, while total concentrations remained relatively stable or even increased slightly. The latter effect resulted from the shrinking water volume and continued low-level inputs from piezometer P4, GW2, and the drainage inlet. Outflows were minimal, with only small exports through GW1/P1.

#### Source-specific inflow patterns of individual pesticides and TPs at LSWB S

While the previous section focused on the overall temporal dynamics of water and pesticide fluxes across hydrological phases, this section examines the inflow loads of individual compounds. The analysis highlights the relative importance of different inflow pathways, including drainage and shallow GW via piezometers and GW pipes. This approach allows for further identifying dominant transport routes and compound-specific behavior that contribute to the contamination of the LSWBs.

**Fig. 7:**
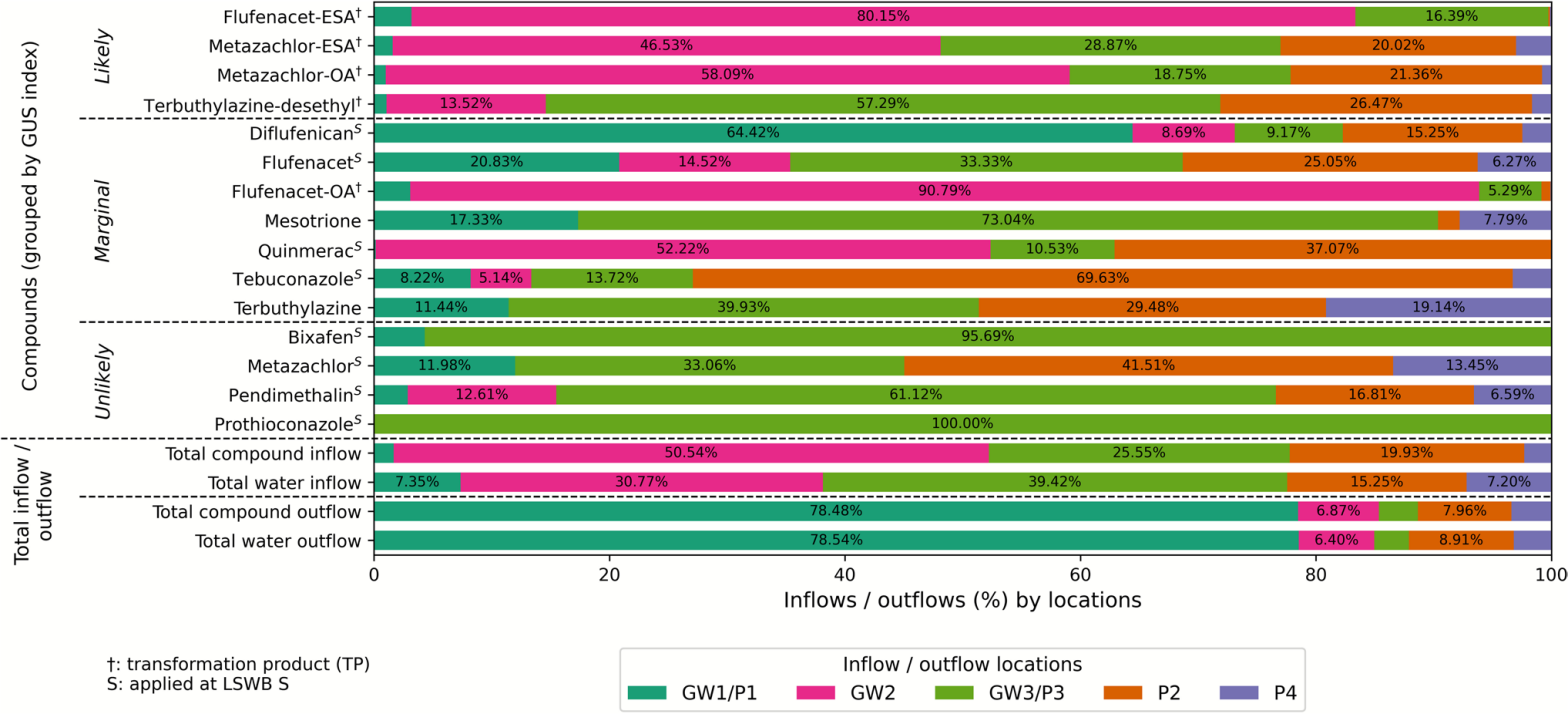
Inflow loads of detected compounds into LSWB S over time, grouped by GUS Index and source. The plot shows the daily inflow loads of detected pesticides and their transformation products (TPs) entering the pond from different inflow sources: shallow groundwater (GW) and piezometers (P). Bars are color-coded by source. Color scheme adapted to LSWB S for visual clarity. Compounds marked with “S” were applied and with “†” indicating TPs. Total inflow and outflow loads and the corresponding water inflow and outflow fluxes are also shown

At LSWB S, pesticide inflow loads were evenly distributed across inflow locations, with key contributions from GW2, GW3/P3, and P2 (Fig. 7). The dominant pathways are GW2, GW3/P3, and P2, each contributing to the overall input. However, the relative pesticide inflow loads do not always correspond to the relative water volumes entering the pond from each source. Only for P2 do compound and water inflows align closely.

No uniform transport pattern was observed across applied compounds. Instead, load distribution reflected compound-specific properties. Flufenacet, despite its moderate mobility, was detected in all inflow sources, showing its ability to reach both shallow and deeper pathways. In contrast, the parent compound metazachlor, with low leaching potential, was primarily detected in piezometers, consistent with limited vertical transport. Their TPs, especially flufenacet-ESA and metazachlor-ESA/OA, were concentrated in GW2, which contributed at least 46% of their total loads. Sorption dominated applied compounds, such as bixafen and pendimethalin, showed highest inputs from GW3/P3, where P3 collects shallow GW near the soil surface, indicating shallow lateral transport. For bixafen, no measurable concentrations were detected in pond water despite its presence in these inflows. In addition, non-applied compounds, such as mesotrione and tebuconazole, were detected across sources but had a pronounced inflow via GW3/P3.

Total inflow quantities into LSWB S were relatively low, despite the application of a greater number of compounds. This is due to the overall lower hydrological connectivity and absence of a drainage system, which limits both inflows and outflows. Nonetheless, certain compounds, such as metazachlor-ESA and -OA, showed very high total input loads of up to 11 g, demonstrating the persistence and mobility of these TPs (Table 5). Importantly, without a connected drainage outlet, pesticide removal from the pond primarily occurs via shallow GW outflow, with GW1/P1 acting as the main export pathway.

**Table 5:** Inflow loads of detected compounds into LSWB S and K over time, grouped by GUS Index. Total inflow and outflow loads and the corresponding water inflow and outflow fluxes are also shown

GUS leaching potential	Pesticide loads by compound and total (mg) and total water flux (m^3^)	LSWB S	LSWB K
**Likely to leach**	Flufenacet-ESA	441.95	1067.45
	Metazachlor-ESA	11,173.30	21,800.93
	Metazachlor-OA	4566.30	7845.07
	Terbuthylazine-desethyl	4.73	3.38
**Marginal leaching potential**	Diflufenican	46.33	297.08
	Flufenacet	5.55	65.66
	Flufenacet-OA	48.59	38.98
	Mesotrione	10.10	432.83
	Quinmerac	88.67	8.77
	Tebuconazole	17.03	64.34
	Terbuthylazine	2.47	143.12
**Unlikely to leachUnlikely to leach**	Bixafen	1.59	22.35
	Metazachlor	11.79	45.44
	Pendimethalin	10.54	5.94
	Prothioconazole	0.10	972.84
	Total compound inflow (mg)	16,429.02	32,814.16
	Total water inflow (m^3^)	2984.11	23,031.17
	Total compound outflow (mg)	−1422.46	−42,836.54
	Total water outflow (m^3^)	−641.49	−21,944.59

#### Source-specific inflow patterns of individual pesticides and TPs at LSWB K

**Fig. 8:**
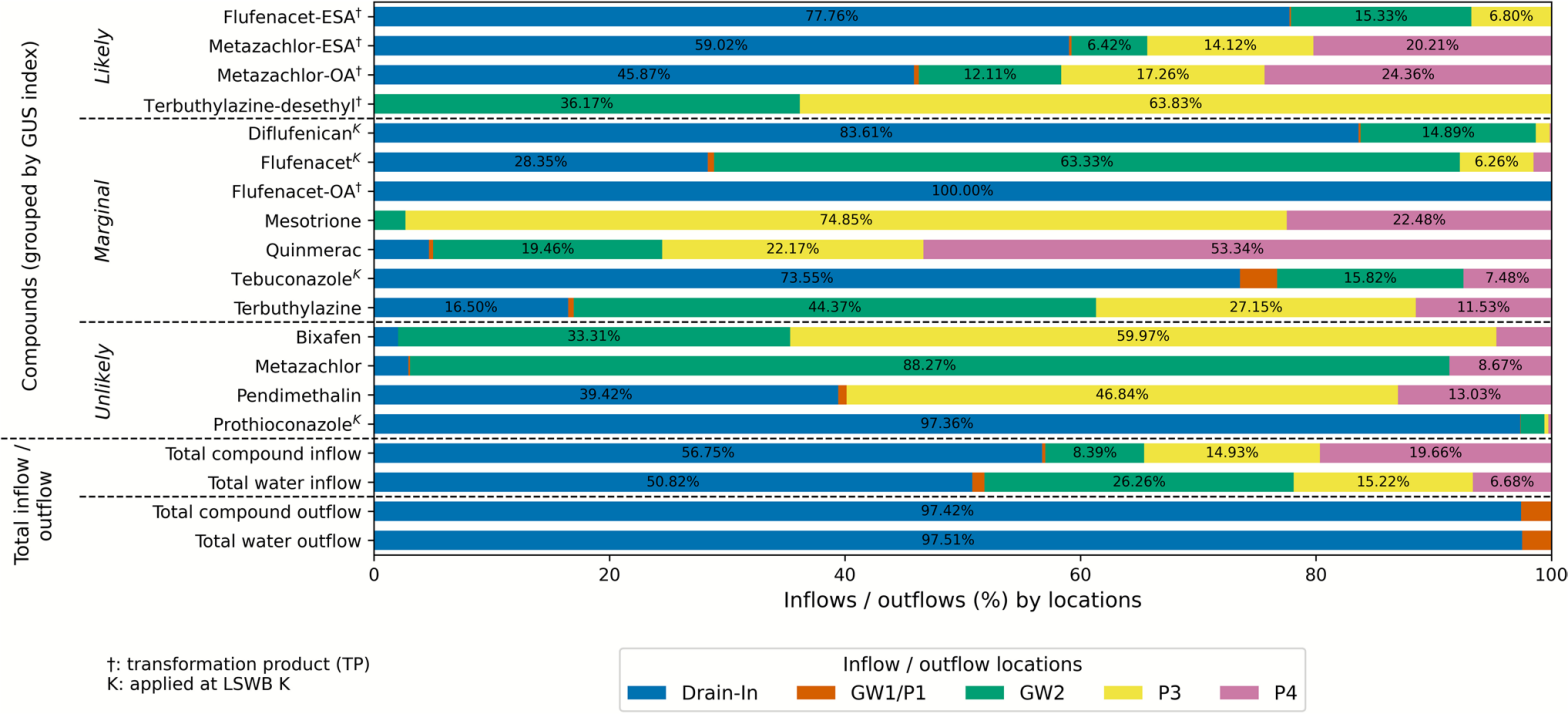
Inflow loads of detected compounds into LSWB K over time, grouped by GUS Index and source. The plot shows the daily inflow loads of detected pesticides and their transformation products (TPs) entering the pond from different inflow sources: shallow groundwater (GW), drainage inlet (Drain In), and piezometers (P). Bars are color-coded by source. Color scheme adapted to LSWB K for visual clarity. Compounds marked with “K” were applied and with “†” indicating TPs. Total inflow and outflow loads and the corresponding water inflow and outflow fluxes are also shown

Compared to LSWB S, total inflow quantities into LSWB K were higher. Across all compounds at LSWB K, the drainage system represents the most important pathway in terms of both water volume and pesticide load. In relative terms, both drainage and P4 delivered disproportionately high pesticide loads compared to their water inflow volumes. By contrast, GW2, while hydraulically important, contributed relatively little to the total compound load (Fig. 8).

The artificial drainage system was the dominant pathway for both water and pesticide loads. For most applied compounds, particularly diflufenican, tebuconazole, and prothioconazole, over 70% of the total inflow load entered via the drainage inlet, due to its direct connection to the treated field and its capacity to rapidly convey pesticide-laden water following application or rainfall. In contrast, the applied and more mobile flufenacet displays a different pattern, with a substantial portion of the inflow, with around 71% entering via the GW, reflecting its leaching potential. For non-applied compounds, the role of shallow GW transport becomes even more pronounced, with compound-specific differences in dominant inflow sources. For instance, terbuthylazine mainly enters the pond via GW2 and P3, while quinmerac shows higher inputs from P4. A particular example is metazachlor, which is mainly transported via the shallow GW across the deeper GW2 pipe, accounting for up to 88% of its total inflow load.

Among the mobile compounds, no consistent trend is observed regarding the dominance of shallow piezometers versus deeper GW pipe inflows. However, for more sorptive compounds such as bixafen and pendimethalin, shallow piezometers contribute more to the inflow, supporting previous findings (“Compound specific contamination patterns across sampling locations” section) that show the limited leachability of these compounds. The highest total inflow loads were observed for the TPs metazachlor-ESA and -OA, followed by flufenacet-ESA and prothioconazole. Notably, despite no recent application of metazachlor, its TPs occurred broadly and at high levels. Despite fewer pesticide applications in the LSWB K catchment, inflow loads to LSWB K were higher, largely driven by the drainage inlet and the overall greater water fluxes (Table 5).

## Discussion

### Spatial variability of pesticide and TP contamination in LSWBs, shallow GW, and drainage

The spatial distribution of pesticide and TP concentrations depended on both compound-specific mobility and site-specific hydrological connectivity. Compounds classified as “likely to leach” were found in high DA across surface water, shallow piezometers, deeper GW pipes, and the drainage inlet at LSWB K. These compounds, primarily the TPs of flufenacet and metazachlor, dominated the compound spectrum. They contributed highly to total concentrations at various times and locations, indicating their strong influence on the overall contamination profile. Their widespread presence underscores their persistence and mobility, even without direct application during the study period. This aligns with findings by Ulrich et al. (2018, 2022), who reported that metazachlor TPs remained detectable in pond water and GW a year after application, whereas the parent compound was mostly confined to the application season. These results indicate that many TPs are more mobile and persistent than their parent compounds, which explains their frequent occurrence in GW-fed systems (Gassmann et al. 2015; Pasquini et al. 2025; Ulrich et al. 2018). In contrast, compounds with high K_foc_ values and low leaching potential, such as pendimethalin and diflufenican, were mainly detected in piezometers near the soil surface, consistent with strong sorption and limited vertical mobility. However, their occasional detection in drainage water at LSWB K suggests that preferential flow and artificial drainage can bypass soil filtration. Petersen et al. (2003) demonstrated that sorbing herbicides, such as pendimethalin, can rapidly reach drainage tiles via macropore flow during early rain events following application.

The detection of both applied and non-applied compounds indicates that LSWBs are influenced not only by local pesticide use but also by legacy inputs and regional transport from other sites. Non-applied residues such as terbuthylazine or mesotrione likely entered via shallow GW from other treated fields. The widespread cultivation of maize in the catchment, where terbuthylazine is commonly applied, further supports the likelihood of regional subsurface transport. Similar patterns have been reported by Lorenz et al. (2025), Slaby et al. (2022), and Ulrich et al. (2022), who found frequent detections of TPs and pesticide residues despite no recent local applications. These observations highlight the role of delayed inputs and long-range subsurface transport in LSWB contamination. In addition, sorption to the soil matrix allows pesticides and TPs to persist in the environment, effectively storing them over extended periods. Under certain hydrological conditions, such as high saturation and increased water fluxes, compounds can desorb and become mobile again. In addition to shallow GW transport, such desorption and subsequent leaching processes can contribute to prolonged contamination of adjacent water bodies (Mamy & Barriuso 2007; Tauchnitz et al. 2020).

Despite the proximity of piezometers and GW pipes, concentrations varied markedly between installations, indicating heterogeneity in shallow GW flow paths. Similar patterns have been observed in other small water bodies. For example, Lischeid & Kalettka (2012) found spatially variable water quality in kettle holes due to discrete inflow sources such as interflow and regional GW. This heterogeneity complicates source attribution, as adjacent sampling points may reflect distinct contaminant pathways. It also emphasizes the strong influence of surrounding arable land use on pesticide delivery (Joniak et al. 2016; Kristensen & Globevnik 2014).

The observed spatial patterns indicate a buffering function of LSWBs. Inflow points generally exhibited higher pesticide concentrations than pond water and outflow zones, suggesting attenuation through dilution, sorption, or degradation processes. This aligns with Imfeld et al. (2021), who described agricultural ponds as biogeochemical hotspots capable of reducing pesticide loads. Likewise, Joffre et al. (2024) and Willkommen et al. (2022) highlighted the retention capacity of small water bodies under agricultural pressure. Internal processes within LSWBs can lower contaminant levels over time (Vymazal & Březinová, 2015). However, residues may be remobilized and continue to flow during fluctuations in hydrological conditions. Thus, LSWBs act as both sinks and potential secondary sources of contamination. Their landscape position and strong interface with agricultural land make them particularly sensitive to both direct and diffuse contamination inputs (Dudzińska et al. 2020; Kristensen & Globevnik 2014; Lischeid et al. 2018).

### Temporal variability of concentrations in the LSWBs and their adjacent compartments

While compound properties and flow pathways largely governed the spatial distribution of pesticides, the temporal dynamics reveal how LSWBs respond to seasonal shifts in land use and hydrology. Concentration peaks occurred primarily in autumn and winter, underscoring the alignment between pesticide application schedules and rainfall-triggered mobilization. These seasonal pulses illustrate the high reactivity of LSWBs to agricultural inputs under variable hydrological conditions.

At LSWB S, concentrations peaked following autumn applications, particularly during phases of rising pond levels. Elevated values in piezometers and GW pipes indicated active transport from surrounding fields, with corresponding increases in inflow loads. Sustained high concentrations in some piezometers and GW pipes suggest long-distance shallow GW transport, highlighting the role of LSWBs as integrators of both local and regional contamination. At LSWB K, concentrations declined after pesticide use ceased, yet drainage inflow and specific GW and piezometer pipes, e.g. GW3, remained contaminated during the study period. In tile-drained systems, short-lived but intense pulses often occur after precipitation events (Blann et al. 2009; Kobierska et al. 2020), which conventional sampling may fail to capture. Betz-Koch et al. (2023) and Ulrich et al. (2018) reported that pesticide concentrations in LSWBs and streams often peaked immediately after rainfall. These first flush events can result in substantial short-term loads and ecotoxicological exposure, underscoring the need for high-frequency monitoring during critical periods. Other studies similarly showed that conventional grab sampling often misses short-lived peaks (Rabiet et al. 2010; Stehle et al. 2013; Szöcs et al. 2017), an issue recognized in LSWB monitoring (Liess et al. 2022; Lorenz et al. 2017).

Beyond these acute peaks, our results also demonstrate persistent low-level contamination. Shallow GW compartments responded quickly to application phases, while deeper GW pipes showed more buffered, long-term profiles. Ulrich et al. (2022) also found TPs to be of high frequency in several LSWBs. Similarly, Le Cor et al. (2021) traced long-term contamination to legacy residues and regional GW flow, showing that LSWBs may receive inputs even without recent local pesticide use. Navarro et al. (2024) further observed that pesticide concentrations in small European water bodies temporally fell below ecotoxicological thresholds but frequently exceeded them following pesticide use. This seasonal pattern reinforces the tight coupling between land use, rainfall, and pesticide contamination risk in LSWBs. Importantly, the co-occurrence of short-term pulses and long-term background contamination poses a dual challenge for risk assessment: episodic events may lead to high and short-term inputs, whereas, for example, persistent TPs contribute to chronic exposure.

### Dominant contamination pathways

The interplay between hydrology, landscape position, and compound-specific properties shaped the transport and fate of pesticides in the two LSWBs. While both systems received inputs from surrounding agricultural land, the dominant contamination pathways differed markedly. At LSWB K, artificial drainage served as a direct and highly dynamic conduit for pesticide transport, bypassing the soil matrix and rapidly delivering both water and solutes to the pond after rainfall. This pathway was especially important during application periods and supports previous observations of macropore-driven bypass flow in tile-drained landscapes (e.g., Lischeid et al. 2023; Petersen et al. 2003; Reichenberger et al. 2007). Even strongly sorbing compounds such as diflufenican and prothioconazole were detected in drainage inflows, underscoring the ability of structured soils to mobilize low-mobility compounds through preferential pathways. Similar transport of glyphosate and pendimethalin via bypass and particulate pathways has been reported in structured, drained soils (Kjær et al. 2011). In contrast, LSWB S, lacking artificial drainage, received pesticides gradually via shallow GW. Inflows were distributed across piezometers and GW pipes. This reflects slower, subsurface-dominated transport from adjacent fields and broader GW flow systems. Similar continuous pesticide inputs via GW were observed in other drainage-free LSWBs (e.g., Lorenz et al. 2025; Ulrich et al. 2018, 2022) and other water bodies (e.g., Belles et al. 2019; Welch et al. 2019), especially where legacy or regional sources dominate.

Compound-specific transport generally followed expectations based on physicochemical properties. Leachable compounds, such as the TPs or quinmerac, occurred mainly in deeper GW, while more sorptive compounds, such as bixafen and pendimethalin, were concentrated in shallow piezometers. However, site-specific factors modified these trends. For instance, bixafen was detected in all compartments at LSWB K, including pond water and 3 m GW pipes, despite the last application in 2018, suggesting either historical accumulation or particle-bound transport. These findings illustrate how compound behavior interacts with local hydrology to shape observed transport pathways. Pesticide export also varied markedly between sites. At LSWB K, the drainage outlet facilitated efficient contaminant removal during wet periods, resulting in a flow-through system with limited retention capacity. Such dynamics are typical of tile-drained ponds with short hydraulic residence times, where pesticide pulses can move rapidly through the system and into downstream waters (Rabiet et al. 2010; Willkommen et al. 2022). By contrast, LSWB S exported pesticides only slowly via shallow GW. This can result in longer contaminant residence times and sustained exposure during periods of low flow. Additionally, these systems may serve as long-term sources of pesticide exposure for connected aquifers or downstream water bodies.

The interplay of compound properties, hydrological pathways, and drainage systems governs the fate of pesticides in LSWBs. The presence or absence of artificial drainage not only determines dominant transport routes but also influences the timing and magnitude of pesticide export. These findings highlight the complexity of pesticide movement into and out of LSWBs, an area still insufficiently addressed by current monitoring schemes (Lorenz et al. 2025; Ulrich et al. 2022). This underscored call under the EU Water Framework Directive and related initiatives to better integrate LSWB into pesticide risk assessment and regulatory frameworks (Liess et al. 2023; Stanković et al. 2023; Weisner et al. 2022).

### Key findings in the context of the research questions

Pesticide and TP concentrations and detection amounts varied considerably across space and time, influenced by compound properties, land management practices, and hydrological conditions. Regarding spatial patterns (RQ1), concentrations differed between LSWBs, shallow GW in piezometers and GW pipes, and drainage. Compounds classified as “likely to leach,” such as metazachlor-ESA, metazachlor-OA, and flufenacet-ESA, were found consistently across all compartments. In contrast, strongly sorbing compounds, such as pendimethalin and diflufenican, were mainly detected in shallow piezometers. This highlights the influence of both compound mobility and flow pathways on spatial distribution. Temporally (RQ2), pesticide and TP concentrations in LSWBs and adjacent compartments peaked during autumn and winter, coinciding with application periods and increased rainfall. Short-lived but high-intensity contamination pulses were observed, particularly at LSWB K due to drainage inflows. Meanwhile, persistent low-level contamination in GW, particularly at LSWB S, reflected ongoing subsurface transport, even in the absence of recent applications. Regarding contamination pathways (RQ3), tile drainage dominated pesticide input and export at LSWB K, facilitating the rapid transfer of both mobile and sorbing compounds. At LSWB S, inputs occurred primarily via shallow and intermediate GW, particularly for persistent TPs. Export routes mirrored inflows: LSWB K functioned as a flow-through system with fast drainage losses, whereas LSWB S retained contaminants longer, releasing them gradually via seepage. Together, these findings demonstrate that LSWB integrates contamination signals from multiple sources and timescales, acting as both sinks and sources depending on local conditions.

### Transferability of findings to other LSWBs

The specific concentration levels and loads derived for the two studied LSWBs are not directly transferable to other systems, as they depend on local hydrology, compound properties, and land management. However, the underlying processes are likely relevant for many LSWBs, which occur worldwide (Holgerson and Raymond 2016; Oertli, 2005), and are indicative of the potential exposure that other LSWBs may experience. Typological studies show that comparable open-water LSWB types occur widely in glacial moraine and lowland landscapes, often influenced by agriculture (Biggs et al. 2005; Kalettka & Rudat 2006; Lorenz et al. 2024). In many of these settings, LSWBs are hydraulically connected to shallow, unconfined aquifers, and several studies have documented a strong influence of shallow GW on water balances (Golus & Bajkiewicz-Grabowska 2017; Lischeid et al. 2017; Vyse et al. 2020). This increases the likelihood that GW acts as an important transport pathway for pesticides and TPs (Lischeid & Kalettka 2012; Lorenz et al. 2025; Ulrich et al. 2018, 2022). For LSWBs that intersect such shallow GW and are embedded in arable land, such as LSWB S, similar inputs of mobile and persistent compounds are therefore likely, even if the dominant compounds differ regionally. This is particularly relevant for frequently used compounds and their TPs with high leaching potential and persistence, such as metazachlor and flufenacet TPs (Liess et al. 2023; Ulrich et al. 2018), as seen for both studied LSWBs.

In addition, large parts of agricultural landscapes in North-east German lowlands and other European plains are artificially drained, and tile drains often discharge directly into ditches and small water bodies (Tetzlaff and Kuhr, 2017); Feick et al. 2005). LSWBs that receive a substantial share of their water balance from such drainage inflows are therefore expected to show contamination patterns similar to LSWB K, with short-lived but high-concentration pulses that reflect the timing of pesticide applications, precipitation, and drainage flow, and that can bypass the buffering capacity of the soil matrix (Belles et al. 2019; Petersen et al. 2003; Reichenberger et al. 2007). Taken together, the two study LSWBs represent endmembers along a gradient from GW-dominated to drainage-dominated systems. Many agricultural LSWBs will fall between these endmembers. For all of them, the combination of seasonal pesticide applications, shallow GW connectivity, and artificial drainage is likely to control both the timing and magnitude of pesticide and TP inputs.

## Conclusions

This study demonstrates that LSWBs in agricultural landscapes are subject to complex contamination dynamics shaped by the properties of pesticides, land use, and hydrological connectivity. By integrating spatial and temporal data from surface water, shallow GW, and drainage systems, we demonstrate that LSWBs function as both recipients and modulators of pesticide and TP inputs. High-resolution monitoring revealed contamination pulses and persistence patterns, while also exposing key limitations in conventional sampling and risk assessment approaches. Current monitoring frameworks often overlook critical transport pathways, particularly subsurface flow and drainage, and tend to neglect persistent TPs, which, in this study, were widespread and long-lasting. Effective pesticide risk assessment must therefore extend beyond surface water snapshots and incorporate long-term, multi-compartment data, especially under dynamic hydrological conditions.

Future research should scale up high-resolution approaches across a range of agro-hydrological settings to assess the generalizability of these patterns. Evaluating the real-world effectiveness of mitigation measures, such as constructed wetlands, buffer zones, or interface solutions, will be essential. Additionally, climate-resilient management strategies are urgently needed, as shifts in precipitation patterns may intensify the mobilization and retention of pesticides. Protecting LSWBs in agricultural catchments requires integrated approaches that reflect their multifunctional roles in water retention, biodiversity support, and contaminant buffering. Because these systems are highly responsive to both land use and hydrology, effective protection demands closer alignment between science, practice, and policy. This includes adapting monitoring strategies to realistic transport dynamics, routinely accounting for TPs, and designing flexible management interventions at both field and catchment scales. Advancing our conceptual understanding alongside practical tools is key to safeguarding these ecologically valuable water bodies in an increasingly dynamic agricultural landscape.

## Supplementary Information

Below is the link to the electronic supplementary material.

ESM 1 (DOCX 4.28 MB)

## Data Availability

The methods and data supporting the results of this study are available from the corresponding author upon reasonable request.
