# Supplementary material for: From field to pond and beyond: bidirectional transport of pesticides and their transformation products in lentic small water bodies in Northern Germany
Source: Environ Sci Pollut Res Int. 2026 Jan 7;33(2):423–41. doi: 10.1007/s11356-025-37317-z (PMC12882944; doi:10.1007/s11356-025-37317-z)
Supplement: Supplementary file 1 — (DOCX 4.28 MB) [file 11356_2025_37317_MOESM1_ESM.docx]

**Supplementary Information**


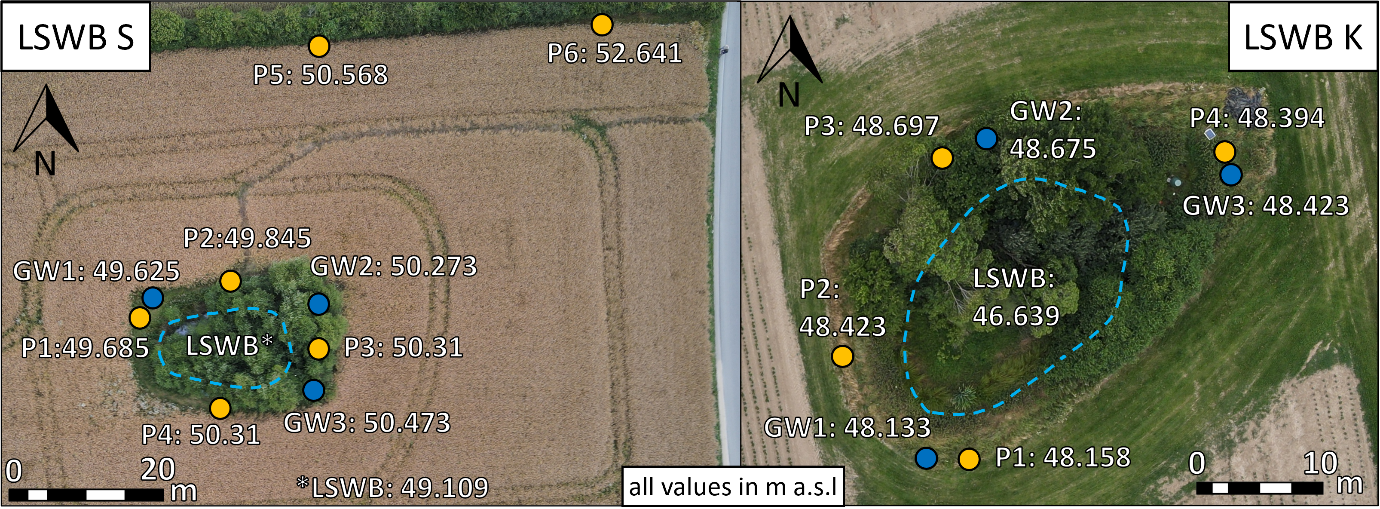


**Fig 9** Aerial images of the two lentic small water bodies (LSWB S and LSWB K) highlight the water areas as well as the piezometers (P), groundwater pipes (GW). All values show the height of each GW and P pipe in meter above sea level (m a.s.l.).


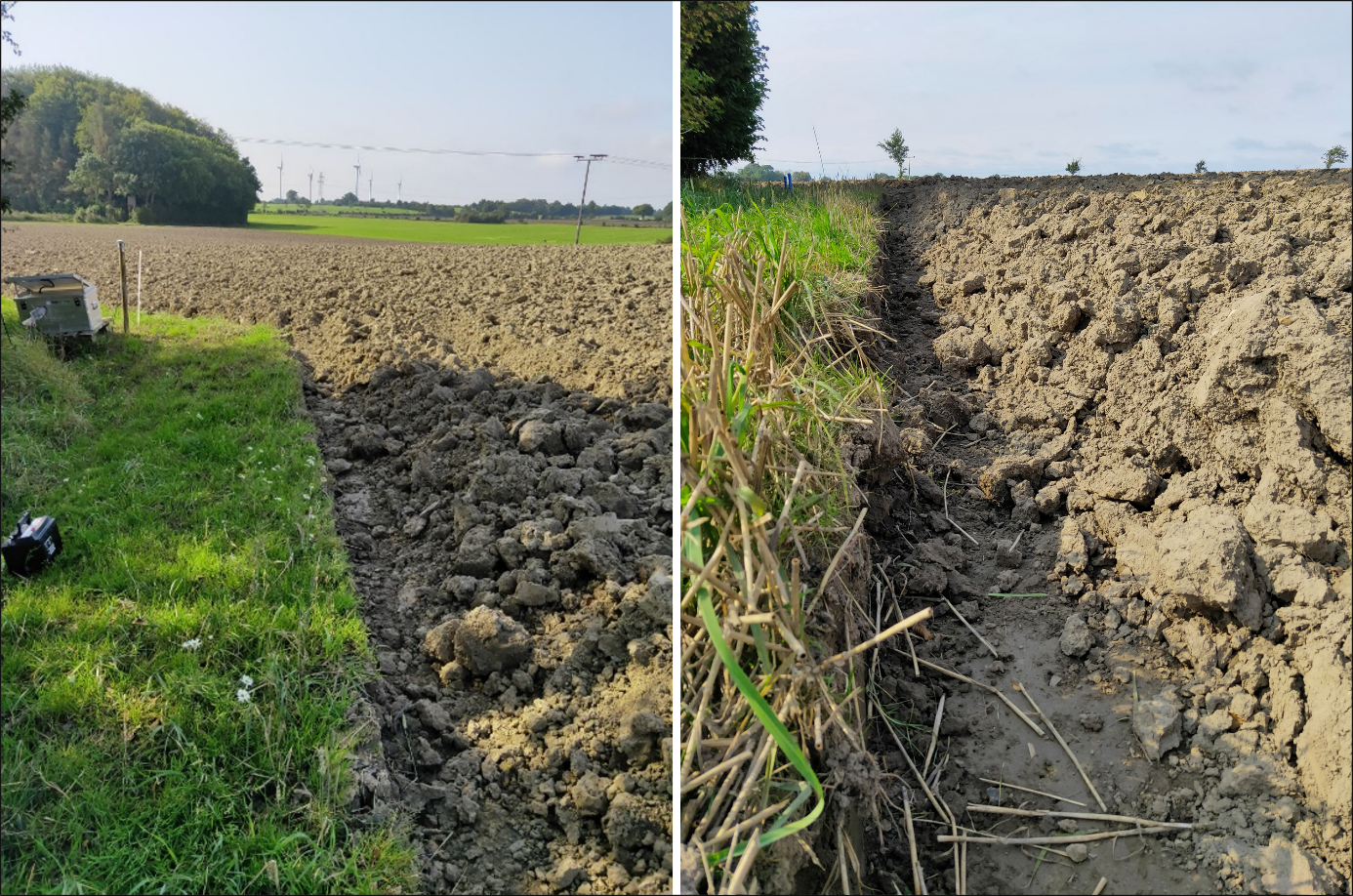


**Fig 10** Photographs of the ploughed furrow at the edge of LSWB S.

**Table 6** Summed inflow and outflow loads at LSWB K with each hydrological phase and the overall loads

|  |  | Filling | Plateau | Drying | Filling | Plateau | Drying | Overall |
| --- | --- | --- | --- | --- | --- | --- | --- | --- |
|  |  | 01.10.20 - 06.11.20 | 07.11.20 - 23.06.21 | 24.06.21 - 29.09.21 | 30.09.21 - 22.10.21 | 23.10.21 - 20.07.22 | 21.07.22 - 31.07.22 | 01.10.20 - 31.07.22 |
| Summed loads inflow (mg) | GW1/P1 | 0.00 | 79.09 | 37.89 | 13.02 | 12.34 | 0.00 | 142.34 |
|  | GW2 | 0.00 | 1658.49 | 81.76 | 0.00 | 1017.69 | 0.14 | 2758.08 |
|  | P3 | 0.00 | 2977.27 | 0.00 | 61.18 | 1864.41 | 0.00 | 4902.86 |
|  | P4 | 478.61 | 2627.76 | 820.06 | 213.87 | 2254.55 | 55.82 | 6450.67 |
|  | Dr | 0.00 | 11870.82 | 83.47 | 153.31 | 6497.36 | 30.02 | 18634.98 |
| Summed loads outflow (mg) | GW1/P1 | -198.28 | -419.59 | -116.33 | 0.00 | -350.64 | -16.19 | -1101.04 |
|  | GW2 | 0.00 | -8.87 | 0.00 | 0.00 | 0.00 | -1.24 | -10.11 |
|  | P3 | 0.00 | 0.00 | 0.00 | 0.00 | 0.00 | 0.00 | 0.00 |
|  | P4 | 0.00 | 0.00 | 0.00 | 0.00 | 0.00 | 0.00 | 0.00 |
|  | Dr | -379.35 | -22234.55 | -246.08 | -519.37 | -18356.67 | -52.22 | -41788.24 |

**Table 7** Summed inflow and outflow loads at LSWB S with each hydrological phase and the overall loads

|  |  | Filling | Drying | Filling | Plateau | Drying | Overall |
| --- | --- | --- | --- | --- | --- | --- | --- |
|  |  | 01.10.20 - 12.05.21 | 13.05.21 - 29.09.21 | 30.09.21 - 22.01.22 | 23.01.22 - 09.04.22 | 10.04.22 - 31.07.22 | 01.10.20 - 31.07.22 |
| Summed loads inflow (mg) | GW1/P1 | 179.11 | 0.00 | 98.39 | 0.00 | 0.00 | 277.50 |
|  | GW2 | 453.33 | 6.03 | 5442.48 | 1637.35 | 765.76 | 8304.93 |
|  | GW3/P3 | 913.35 | 34.45 | 1097.08 | 1472.01 | 741.29 | 4258.19 |
|  | P2 | 456.01 | 186.48 | 2389.34 | 224.72 | 23.20 | 3279.74 |
|  | P4 | 53.60 | 1.78 | 110.68 | 162.68 | 50.33 | 379.06 |
| Summed loads outflow (mg) | GW1/P1 | -79.18 | -321.59 | -118.11 | -223.19 | -493.15 | -1235.22 |
|  | GW2 | 0.00 | -7.98 | 0.00 | 0.00 | -95.75 | -103.73 |
|  | GW3/P3 | 0.00 | -0.73 | 0.00 | 0.00 | -48.62 | -49.35 |
|  | P2 | -3.25 | -31.03 | -6.24 | -2.34 | -85.82 | -128.67 |
|  | P4 | 0.00 | -20.40 | 0.00 | 0.00 | -31.42 | -51.82 |

**Table 8** Limit of detection (LOD), limit of quantification (LOQ) of all analyzed compounds

| Compound | Limit of detection (LOD)  (µg L^-1^) | Limit of quantification (LOQ)  (µg L^-1^) |
| --- | --- | --- |
| Bixaphen | 0.0015 | 0.005 |
| D5-Terbuthylazin | 0.02 | 0.005 |
| D6-Metazachlor-ESA | 0.025 | 0.075 |
| Diflufenican | 0.0015 | 0.005 |
| Dimethenamid | 0.0015 | 0.005 |
| Florasulam | 0.003 | 0.005 |
| Flufeancet | 0.0015 | 0.005 |
| Flufenacet-ESA | 0.0075 | 0.025 |
| Flufenacet-OA | 0.0075 | 0.025 |
| Fluroxypyr | 0.025 | 0.075 |
| Mefenpyr | 0.02 | 0.005 |
| Mesosulfuron | 0.0075 | 0.01 |
| Mesotrione-pos | 0.025 | 0.075 |
| Metazachlor | 0.0015 | 0.01 |
| Metazachlor-ESA-pos | 0.025 | 0.075 |
| Metazachlor-OA-pos | 0.025 | 0.075 |
| Metconazol | 0.02 | 0.01 |
| Metolachlor | 0.0075 | 0.025 |
| Metrafenone | 0.0075 | 0.025 |
| Norflurazon | 0.0015 | 0.005 |
| Pendimethalin | 0.003 | 0.01 |
| Pirimicarb | 0.0015 | 0.005 |
| Propoxycarbazone | 0.025 | 0.075 |
| Prothioconazol | 0.003 | 0.01 |
| Quinmerac | 0.002 | 0.005 |
| Tebuconazol | 0.0015 | 0.005 |
| Terbuthylazin | 0.0015 | 0.005 |
| Terbuthylazin-desethyl | 0.0015 | 0.005 |
| Trichlopyr | 0.025 | 0.075 |

**Table 9** Quality control for all analyzed compounds with reproducibility (%, periodically measured standard solutions), recovery (%, analysis of recovery from spiked samples) and repeatability (%, analysis of multiple standard solutions sequentially)

| Compound | Reproducibility % | Recovery % | Repeatability % |
| --- | --- | --- | --- |
| Bixafen | 90 | 65 | 95 |
| D5-Terbuthylazine | 89 | 80 | 94 |
| D6-Metazachlor-ESA | 87 | 90 | 92 |
| Diflufenican | 91 | 61 | 96 |
| Dimethenamid | 92 | 62 | 97 |
| Florasulam | 93 | 94 | 98 |
| Flufeancet | 90 | 59 | 96 |
| Flufenacet-ESA | 89 | 73 | 94 |
| Flufenacet-OA | 86 | 83 | 94 |
| Fluroxypyr | 91 | 73 | 96 |
| Mefenpyr | 86 | 58 | 91 |
| Mesosulfuron | 93 | 80 | 98 |
| Mesotrione | 77 | 93 | 82 |
| Metazachlor | 93 | 58 | 99 |
| Metazachlor-ESA | 89 | 76 | 94 |
| Metazachlor-OA | 84 | 63 | 93 |
| Metconazole | 91 | 69 | 97 |
| Metolachlor | 90 | 68 | 96 |
| Metrafenone | 92 | 69 | 97 |
| Norflurazon | 89 | 57 | 94 |
| Pendimethalin | 85 | 61 | 90 |
| Pirimicarb | 46 | 48 | 65 |
| Propoxycarbazone | 87 | 80 | 92 |
| Prothioconazole | 88 | 59 | 93 |
| Quinmerac | 91 | 56 | 97 |
| Tebuconazole | 92 | 68 | 98 |
| Terbuthylazin | 92 | 63 | 98 |
| Terbuthylazin-desethyl | 93 | 57 | 98 |
| Triclopyr | 89 | 33 | 95 |

**Table 10** Gradient elution with eluent A being ultra-pure water, modified with 0.005% formic acid and 2.5 mmol/L ammonium formate, and eluent B being pure MeOH

| Time (min) | Eluent A | Eluent B | Flow (mL/min) |
| --- | --- | --- | --- |
| 0.00 | 98.00 | 2.00 | 0.60 |
| 1.00 | 98.00 | 2.00 | 0.60 |
| 2.00 | 50.00 | 50.00 | 0.60 |
| 7.50 | 10.00 | 90.00 | 0.60 |
| 10.50 | 2.00 | 98.00 | 0.60 |
| 13.00 | 2.00 | 98.00 | 0.60 |

**Table 11** Source and iFunnel parameters of the analysis method

| Parameter | Value | Unit |
| --- | --- | --- |
| Gas temperature | 200 | °C |
| Gas flow | 13 | L/min |
| Nebulizer | 40 | Psi |
| Sheath gas temperature | 375 | °C |
| Sheath gas flow | 12 | L/min |
| Capillary positive | 3500 | V |
| Capillary positive | 3000 | V |
| Nozzle Voltage positive | 0 | V |
| Nozzle Voltage negative | 500 | V |
| High pressure RF pos. | 150 | V |
| High pressure RF neg. | 90 | V |
| Low pressure RF | 80 | V |
| Low pressure RF | 60 | V |

**Table 12** All analyzed compounds with precursor ions, product ions, retention times (Ret Time), delta retention times (Delta Ret Time), Fragmentor setting, collison energy, cell accelerator voltage and polarity

| Compound Name | Precursor Ion | Product Ion | Ret Time (min) | Delta Ret Time (min) | Fragmentor | Collision Energy | Cell Accelerator Voltage | Polarity |
| --- | --- | --- | --- | --- | --- | --- | --- | --- |
| Bixafen | 414.1 | 394.1 | 9.1 | 1.75 | 380 | 15 | 3 | Positive |
| Bixafen | 414.1 | 374.1 | 9.1 | 1.75 | 380 | 25 | 3 | Positive |
| Bixafen | 414.1 | 266.1 | 9.1 | 1.75 | 380 | 30 | 3 | Positive |
| D5-Terbuthylazine | 235.2 | 179 | 8.5 | 1.75 | 380 | 20 | 3 | Positive |
| D5-Terbuthylazine | 235.2 | 137 | 8.5 | 1.75 | 380 | 28 | 3 | Positive |
| D5-Terbuthylazine | 235.2 | 69 | 8.5 | 1.75 | 380 | 46 | 3 | Positive |
| D6-Metazachlor-ESA | 328 | 248 | 5.6 | 1.75 | 380 | 22 | 3 | Negative |
| D6-Metazachlor-ESA | 328 | 154 | 5.6 | 1.75 | 380 | 24 | 3 | Negative |
| D6-Metazachlor-ESA | 328 | 120.4 | 5.6 | 1.75 | 380 | 28 | 3 | Negative |
| Diflufenican | 395 | 266 | 9.9 | 1.75 | 380 | 30 | 3 | Positive |
| Diflufenican | 395 | 246 | 9.9 | 1.75 | 380 | 45 | 3 | Positive |
| Dimethenamid | 276 | 244 | 8.4 | 1.75 | 380 | 15 | 3 | Positive |
| Dimethenamid | 276 | 168 | 8.4 | 1.75 | 380 | 25 | 3 | Positive |
| Florasulam | 360.1 | 192.1 | 5.7 | 1.75 | 380 | 20 | 3 | Positive |
| Florasulam | 360.1 | 129.1 | 5.7 | 1.75 | 380 | 35 | 3 | Positive |
| Flufeancet | 364.1 | 194.2 | 8.8 | 1.75 | 380 | 10 | 3 | Positive |
| Flufeancet | 364.1 | 152.1 | 8.8 | 1.75 | 380 | 20 | 3 | Positive |
| Flufenacet-ESA | 273.9 | 120.7 | 5.8 | 1.75 | 380 | 22 | 3 | Negative |
| Flufenacet-ESA | 273.9 | 79.7 | 5.8 | 1.75 | 380 | 40 | 3 | Negative |
| Flufenacet-OA | 223.9 | 151.8 | 5.7 | 1.75 | 380 | 8 | 3 | Negative |
| Flufenacet-OA | 223.9 | 135.8 | 5.7 | 1.75 | 380 | 24 | 3 | Negative |
| Fluroxypyr | 253.1 | 233 | 5.7 | 1.75 | 380 | 6 | 3 | Negative |
| Fluroxypyr | 253.1 | 194.9 | 5.7 | 1.75 | 380 | 14 | 3 | Negative |
| Mefenpyr | 373 | 327.1 | 9.5 | 1.75 | 380 | 10 | 3 | Positive |
| Mefenpyr | 373 | 299.1 | 9.5 | 1.75 | 380 | 30 | 3 | Positive |
| Mefenpyr | 373 | 159.8 | 9.5 | 1.75 | 380 | 40 | 3 | Positive |
| Mesosulfuron | 504 | 182.1 | 7.2 | 1.75 | 380 | 34 | 3 | Positive |
| Mesosulfuron | 504 | 162.2 | 7.2 | 1.75 | 380 | 50 | 3 | Positive |
| Mesotrione | 340 | 228 | 5 | 1.75 | 380 | 17 | 3 | Positive |
| Mesotrione | 340 | 104 | 5 | 1.75 | 380 | 28 | 3 | Positive |
| Metazachlor | 278.1 | 210 | 7.5 | 1.75 | 380 | 10 | 3 | Positive |
| Metazachlor | 278.1 | 134 | 7.5 | 1.75 | 380 | 25 | 3 | Positive |
| Metazachlor | 278.1 | 105.1 | 7.5 | 1.75 | 380 | 50 | 3 | Positive |
| Metazachlor-ESA | 324.1 | 256.1 | 5.3 | 1.75 | 380 | 8 | 3 | Positive |
| Metazachlor-ESA | 324.1 | 134.1 | 5.3 | 1.75 | 380 | 30 | 3 | Positive |
| Metazachlor-OA | 274 | 162 | 5.3 | 1.75 | 380 | 7 | 3 | Positive |
| Metazachlor-OA | 274 | 134 | 5.3 | 1.75 | 380 | 18 | 3 | Positive |
| Metconazole | 320.2 | 125 | 9.6 | 1.75 | 380 | 55 | 3 | Positive |
| Metconazole | 320.2 | 70.1 | 9.6 | 1.75 | 380 | 30 | 3 | Positive |
| Metolachlor | 284 | 252 | 9.1 | 1.75 | 380 | 15 | 3 | Positive |
| Metolachlor | 284 | 176 | 9.1 | 1.75 | 380 | 30 | 3 | Positive |
| Metrafenone | 409 | 226.9 | 9.8 | 1.75 | 380 | 25 | 3 | Positive |
| Metrafenone | 409 | 209.1 | 9.8 | 1.75 | 380 | 20 | 3 | Positive |
| Norflurazon | 304 | 283.7 | 7.8 | 1.75 | 380 | 26 | 3 | Positive |
| Norflurazon | 304 | 160 | 7.8 | 1.75 | 380 | 38 | 3 | Positive |
| Norflurazon | 304 | 140 | 7.8 | 1.75 | 380 | 46 | 3 | Positive |
| Pendimethalin | 282.1 | 212.1 | 11.1 | 1.75 | 380 | 10 | 3 | Positive |
| Pendimethalin | 282.1 | 194.1 | 11.1 | 1.75 | 380 | 20 | 3 | Positive |
| Pirimicarb | 239.2 | 182 | 7.8 | 1.75 | 380 | 15 | 3 | Positive |
| Pirimicarb | 239.2 | 72 | 7.8 | 1.75 | 380 | 35 | 3 | Positive |
| Propoxycarbazone | 443 | 202.1 | 5.7 | 1.75 | 380 | 18 | 3 | Positive |
| Propoxycarbazone | 443 | 159 | 5.7 | 1.75 | 380 | 40 | 3 | Positive |
| Prothioconazole | 342.1 | 306 | 9.5 | 1.75 | 380 | 14 | 3 | Negative |
| Prothioconazole | 342.1 | 100 | 9.5 | 1.75 | 380 | 24 | 3 | Negative |
| Prothioconazole | 342.1 | 35.1 | 9.5 | 1.75 | 380 | 30 | 3 | Negative |
| Quinmerac | 222 | 204.1 | 5.2 | 1.75 | 380 | 23 | 3 | Positive |
| Quinmerac | 222 | 141 | 5.2 | 1.75 | 380 | 43 | 3 | Positive |
| Tebuconazole | 308.2 | 125 | 9.3 | 1.75 | 380 | 50 | 3 | Positive |
| Tebuconazole | 308.2 | 70.2 | 9.3 | 1.75 | 380 | 25 | 3 | Positive |
| Terbuthylazin | 230 | 174 | 8.5 | 1.75 | 380 | 20 | 3 | Positive |
| Terbuthylazin | 230 | 132 | 8.5 | 1.75 | 380 | 30 | 3 | Positive |
| Terbuthylazin-desethyl | 202.2 | 146.2 | 7.1 | 1.75 | 380 | 20 | 3 | Positive |
| Terbuthylazin-desethyl | 202.2 | 110.3 | 7.1 | 1.75 | 380 | 26 | 3 | Positive |
| Terbuthylazin-desethyl | 202.2 | 104 | 7.1 | 1.75 | 380 | 34 | 3 | Positive |
| Triclopyr | 256 | 198 | 7.5 | 1.75 | 380 | 8 | 3 | Negative |
| Triclopyr | 254 | 218 | 7.5 | 1.75 | 380 | 1 | 3 | Negative |
| Triclopyr | 254 | 196 | 7.5 | 1.75 | 380 | 8 | 3 | Negative |
